# Is pembrolizumab as safe and efficient as neoadjuvant treatment in localized dMMR/MSI esophagogastric cancers? Results from the IMHOTEP phase II study

**DOI:** 10.1016/j.esmoop.2026.108343

**Published:** 2026-09-09

**Authors:** C. Coutzac, A. Zaanan, A. de Montfort, E. Soularue, R. Cohen, S. Le Sourd, G. Piessen, V. Hautefeuille, M. Ben Abdelghani, E. Blanc, M. Svrcek, E. Samalin, D. Pérol, C. de la Fouchardière

**Affiliations:** 1 Department of Medical Oncology, Centre Léon Bérard, Lyon, France; 2 Department of Gastroenterology and Digestive Oncology, Hôpital Européen Georges Pompidou, Paris University, Assistance Publique-Hôpitaux de Paris, Paris, France; 3 Clinical Research and Innovation Department, Centre Léon Bérard, Lyon, France; 4 Department of Oncology, Institute Mutualiste Montsouris, Paris, France; 5 Department of Medical Oncology, Sorbonne University, Hôpital Saint-Antoine, AP-HP, Paris, France; 6 INSERM UMRS 938, Équipe Instabilité des Microsatellites et Cancer, Équipe Labellisée par la Ligue Nationale Contre le Cancer, SIRIC CURAMUS, Centre de recherche Saint-Antoine, Paris, France; 7 Medical Oncology, Centre Eugène Marquis, Rennes, France; 8 University of Lille, CNRS, Inserm, Chu Lille, UMR9020-U1277 – CANTHER – Cancer Heterogeneity Plasticity and Resistance to Therapies, Lille, France; 9 Department of Hepato-Gastroenterology and Gastrointestinal Oncology, CHU d’Amiens, Amiens, France; 10 Department of Medical Oncology, Paul Strauss Center, Strasbourg, France; 11 Department of Medical Oncology, Institut Régional du Cancer de Montpellier (ICM), Université Montpellier, Montpellier, France; 12 Medical Oncology Department, Paoli-Calmettes Institute, Aix-Marseille University, Marseille, France

**Keywords:** esophagogastric cancer, microsatellite instability, mismatch repair deficiency, pathological complete response, pembrolizumab, perioperative immunotherapy

## Abstract

**Background:**

Patients with localized mismatch repair deficient (dMMR)/microsatellite instability (MSI)-high esophagogastric adenocarcinomas are rare and derive limited benefit from standard perioperative chemotherapy. Immune checkpoint inhibitors have demonstrated efficacy in metastatic dMMR/MSI tumors, and recent phase II studies have shown promising activity in localized stages.

**Patients and methods:**

The multicenter, phase II trial IMHOTEP (NCT04795661) assessed perioperative pembrolizumab in nonmetastatic dMMR/MSI solid tumors. The esophagogastric cohort included patients with localized (cT2-4N0/N+M0) esophagogastric cancer and confirmed dMMR/MSI status. Patients received one or two infusions of neoadjuvant pembrolizumab (400 mg every 6 weeks) and optional post-operative pembrolizumab for up to nine cycles and were candidates for surgery. The primary endpoint was pathological complete response (pCR; ypT0N0).

**Results:**

Between May 2021 and February 2025, among the 41 patients treated with pembrolizumab, 36 received one to two neoadjuvant pembrolizumab infusions and were eligible for surgery and included in the efficacy analysis (surgery: *N* = 27; no surgery: *N* = 9). Of note, five patients were excluded from efficacy analysis (inclusion/exclusion criteria not met, *N* = 3; >2 neoadjuvant pembrolizumab: *N* = 2). The subgroup of operated patients had R0 resection (*N* = 27), and four (14.8%) achieved pCR. With a median follow-up of 25.8 months, 25 (93%) patients experienced no progression, recurrence, or death. Grade ≥3 treatment-related adverse events occurred in nine (22%) patients.

**Conclusions:**

Perioperative pembrolizumab is feasible and safe in localized dMMR/MSI esophagogastric cancers. Further investigation is warranted to optimize the duration and nature of neoadjuvant immunotherapy.

## Highlights

• Localized dMMR/MSI esophagogastric cancer patients received peri-operative pembrolizumab (1-2 cy. before surgery, 9 cy. max after.
• Neoadjuvant pembrolizumab demonstrated a 14.8% pCR rate.
• The 24-month EFS rate was 93% in patients who underwent surgery.
• No new safety signal with pembrolizumab was reported.

## Introduction

Microsatellite instability (MSI) and mismatch repair deficiency (dMMR) are present in ∼5% to 22% of esophagogastric adenocarcinomas (EGAs), and these tumors have consistently shown poorer response to conventional cytotoxic chemotherapy than mismatch repair proficient/microsatellite stable (MSS) tumors in both early and advanced settings.^1^, ^2^, ^3^ Conversely, dMMR/MSI tumors are characterized by a high tumor mutational burden [frameshift mutations (indels)] and immune infiltration, making them sensitive to immune checkpoint inhibitors (ICIs).^4^ Clinical benefit with ICIs has been well established in metastatic dMMR/MSI colorectal cancers (CRCs), with two large randomized phase III trials showing substantial benefit with either programmed cell death protein 1 (PD-1) inhibitor monotherapy (pembrolizumab, nivolumab) or the combination of PD-1 (nivolumab) and cytotoxic T-lymphocyte associated protein 4 (CTLA-4) inhibitors (ipilimumab) in comparison with standard of care.^5^^,^^6^ Furthermore, in localized CRC, several phase II trials have demonstrated an impressive efficacy of ICI as neoadjuvant treatment.^7^, ^8^, ^9^, ^10^ Nonrandomized phase II trials such as NEONIPIGA and INFINITY have extended this concept to localized dMMR/MSI gastric and esophageal cancers, showing pathological complete response (pCR) rates as high as 60% with dual CTLA-4 and PD-1 ICI regimens.^11^^,^^12^

The IMHOTEP trial was designed to evaluate a pragmatic, single-agent perioperative ICI approach using pembrolizumab.^13^ We present results from the EGA cohort, aiming to assess feasibility, efficacy, and safety of perioperative pembrolizumab monotherapy.

## Patients and methods

IMHOTEP is a multicenter, open-label, four-cohort (colorectal, esophagogastric, endometrial, or other digestive cancers) phase II trial (NCT04795661).^13^ Eligible patients were adults with histologically confirmed, locally advanced, nonmetastatic dMMR/MSI adenocarcinoma. The esophagogastric cohort included patients with esophageal, gastroesophageal junction, or gastric adenocarcinoma (cT2-T4 N0M0 or any cT N+M0) based on endoscopy, computed tomography imaging, and/or endoscopic ultrasound. All tumors were confirmed as dMMR/MSI using both immunohistochemistry and molecular MSI status determination based on PCR or next-generation sequencing, as proposed in the current routine practice in France, and recommended by European Society for Medical Oncology guidelines. Additional eligibility criteria included an Eastern Cooperative Oncology Group performance status (ECOG-PS) of 0-1; adequate bone marrow, hepatic, and renal organ functions; and willingness and ability to comply with scheduled visits, treatment plans, laboratory tests, and other study procedures. All inclusion/exclusion criteria are presented in Supplementary Methods S1 (available at https://doi.org/10.1016/j.esmoop.2026.108343). Eligible patients were candidates for surgery.

The decision to administer either one or two cycles of neoadjuvant pembrolizumab (400 mg flat dose every 6 weeks) to achieve appropriate tumor regression before surgery was left to the discretion of each investigator. Surgery was carried out according to institutional standards. Post-operative pembrolizumab was planned for all patients and could be administered to complete nine total cycles, based on post-operative staging, ECOG-PS, and tumor response. Adjuvant chemotherapy or radiotherapy was considered in the cases of poor clinical response, limited tumor shrinkage (including high pathological TNM stage on the surgical specimen), or poor tolerance to pembrolizumab.

The primary endpoint was pCR (ypT0N0). Pathological responses were locally assessed. No assessments were centrally reviewed.

This basket trial used a sequential Bayesian design to evaluate pCR rate, allowing continuous monitoring for efficacy and early discontinuation in case of futility. To reach a sample size of 30 patients in each cohort, the study planned a total sample size of 160 patients, anticipating for the primary endpoint a 25% rate of nonassessable patients owing to surgery refusal.^13^ The primary outcome was pCR (ypT0N0), sequentially analyzed in each cohort with interim analysis planned when 10 patients achieved a 6-week follow-up. The probability of success (pCR) was estimated using a beta-binomial model. The absence of a predefined response rate led to a noninformative prior [beta (1,1)] as pCR initial probability distribution. After each interim analysis, the posterior distributions were updated, and a futility rule recommended enrollment discontinuation in the case of high probability (≥90%) of achieving pCR ≤50%. Enrollment continued until the predefined futility criterion was met or the maximum planned sample size was reached. Final Bayesian estimations of pCR, along with the associated 95% credible interval, and final posterior probability that the estimated pCR was >50% allowed to determine whether the proposed strategy warranted further investigation.

Efficacy analyses for the primary endpoint analysis were conducted in the efficacy-assessable population including patients without major protocol deviations treated with one or two cycles of neoadjuvant pembrolizumab, who underwent surgery. Safety population considered all participants treated with at least one cycle of treatment.

Secondary endpoints included event-free survival (EFS), with event defined as a documented progression (before surgery for a patient having had surgery), a recurrence (after surgery), or death, whichever occurred first; censures for patients alive without disease recurrence or progression; safety (National Cancer Institute Common Terminology Criteria for Adverse Events version 5.0, NCI, Bethesda, MD) including treatment-related (Tr) adverse events (AEs). SAS software version 9.4 (SAS Institute Inc., Cary, NC) was used for all analyses.

## Results

**Figure 1:**
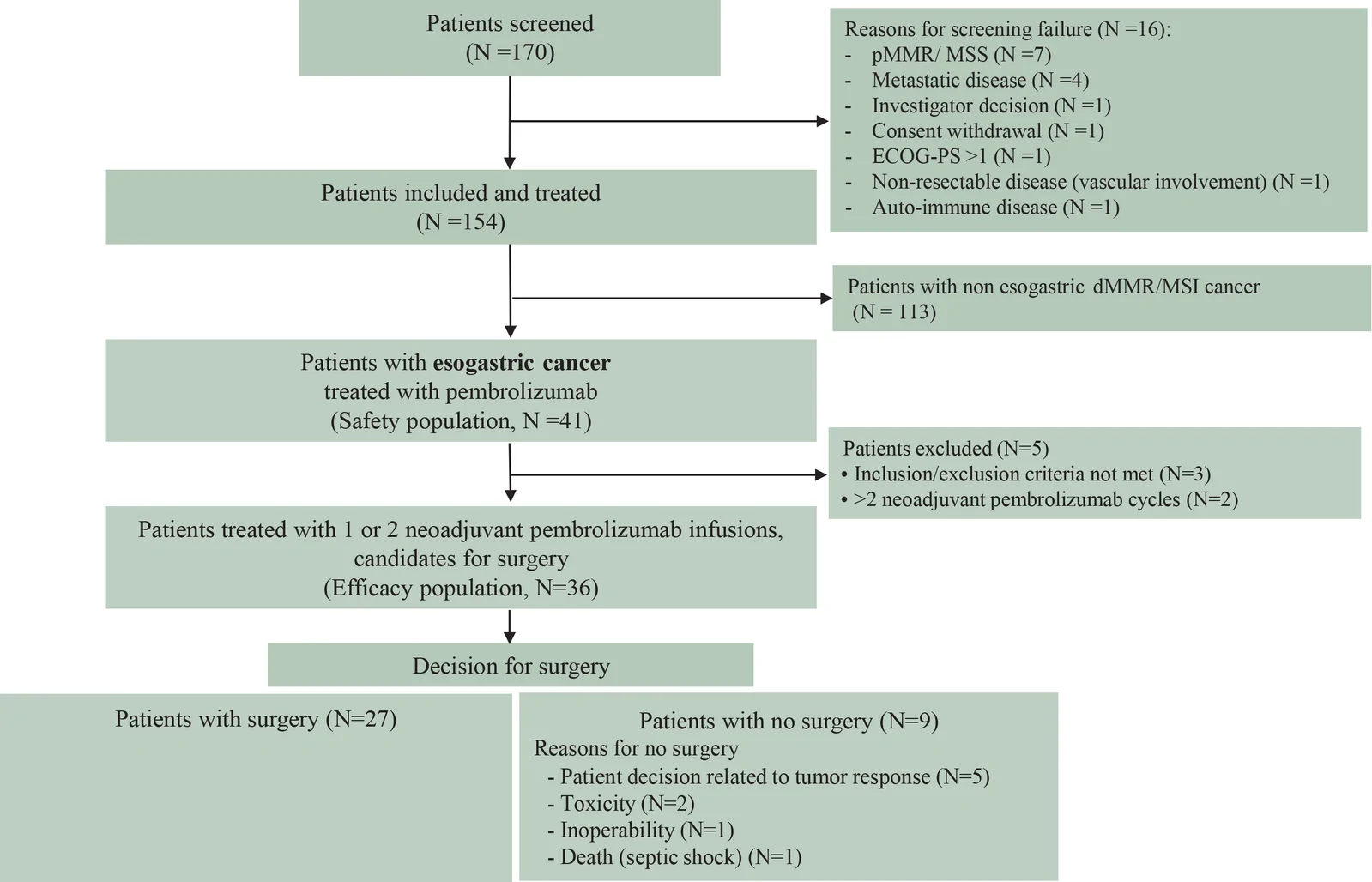
**Study flowchart.** dMMR, mismatch repair deficient; ECOG-PS, Eastern Cooperative Oncology Group performance status; MSI, microsatellite instability; MSS, microsatellite stable; pMMR, mismatch repair proficient.

A total of 41 patients with dMMR/MSI EGA were enrolled between May 2021 and February 2025. Median age was 74 (53-88) years, 61.0% had an ECOG-PS 1, and there was a male predominance (59%) (Table 1). Tumors were mostly located in the stomach (*N* = 24; 58.5%), locally advanced (≥ clinical T3 stage: 78%; clinical positive N stage: 61%), and exhibited mutL homolog 1 (*MLH1*) loss (*N* = 39, 95.1%; loss of *MLH1* and PMS1 homolog 2, mismatch repair system component: *N* = 37; complex loss of MMR including *MLH1*: *N* = 2). Five (12.2%) patients were excluded from the efficacy population because inclusion or exclusion criteria were not met (*N* = 3) or because more than two neoadjuvant pembrolizumab infusions had been received before surgery decision (*N* = 2). Therefore, the efficacy population consisted of 36 patients. Among these 36 patients, 27 underwent surgery after one or two pembrolizumab infusions and were assessable for the primary endpoint (Figure 1).

**Table 1:** Patient and tumor characteristics

Baseline characteristics	*N* = 41
Primary location	
Esophagus	5 (2.4%)
Esophagogastric junction	12 (29.3%)
Stomach	24 (22.0%)
Age (years)	74.0 (53.0-88.0)
Male	24 (58.5%)
ECOG-PS	
0	16 (39.0%)
1	25 (61.0%)
Lynch syndrome	3 (7.3%)
MMR protein expression (IHC)	
Loss of *MLH1* and *PMS2*	37 (90.2%)
Loss of *MSH2* and *MSH6*	1 (2.4%)
Complex loss of MMR	2 (4.9%)
Epstein–Barr virus positive	1 (2.4%)
Clinical T stage at baseline	
T2	9 (22.0%)
T3	29 (70.7%)
T4	3 (7.3%)
Clinical N stage at baseline	
N0	13 (31.7%)
N+	25 (61.0%)
Nx	3 (7.3%)

Data are *n* (%) and median (min–max).

ECOG-PS, Eastern Cooperative Oncology Group performance status; IHC, immunohistochemistry; *MLH1*, mutL homolog 1; MMR, mismatch repair; *MSH2*, mutS homolog 2; *MSH6*, mutS homolog 6; *PMS2*, PMS1 homolog 2, mismatch repair system component.

All 27 patients had complete (R0) resections after neoadjuvant pembrolizumab infusions (one infusion, *N* = 13; two infusions, *N* = 14). The median number of perioperative pembrolizumab infusions was 6 (1-11). pCR was achieved in four (14.8%) patients, with a Bayesian estimation of 17.2% [95% confidence interval (CI) 6.1% to 32.7%] (after one neoadjuvant pembrolizumab infusion: *N* = 1; after two neoadjuvant pembrolizumab infusions: *N* = 3).

A total of 17 patients received post-operative pembrolizumab. The 10 remaining patients included 5 patients with ypN+ disease who received adjuvant chemotherapy [FOLFOX (*N* = 3; stage pT4aN1, *N* = 1); pT3N1 (*N* = 2), FOLFIRINOX (*N* = 1, stage pT3N2), and FLOT (*N* = 1, stage pT3N2)], and 5 other patients who received no adjuvant treatment. The reasons for no surgery in nine patients were as follows: patients’ decision motivated by complete or partial clinical response (*N* = 5) (complete response cT0N0, *N* = 1; partial response, *N* = 2; stable disease, *N* = 2 according to RECIST), toxicity (Tr-nephritis, *N* = 1; Tr-pneumopathy, *N* = 1), inoperability (*N* = 1), and death (septic shock, *N* = 1). These nine patients had received a median number of 4 (1-10) pembrolizumab infusions. Of note, two patients received palliative radiation (median dose = 20 Gy) consecutively to disease progression after one and two pembrolizumab cycles, respectively.

**Figure 2:**
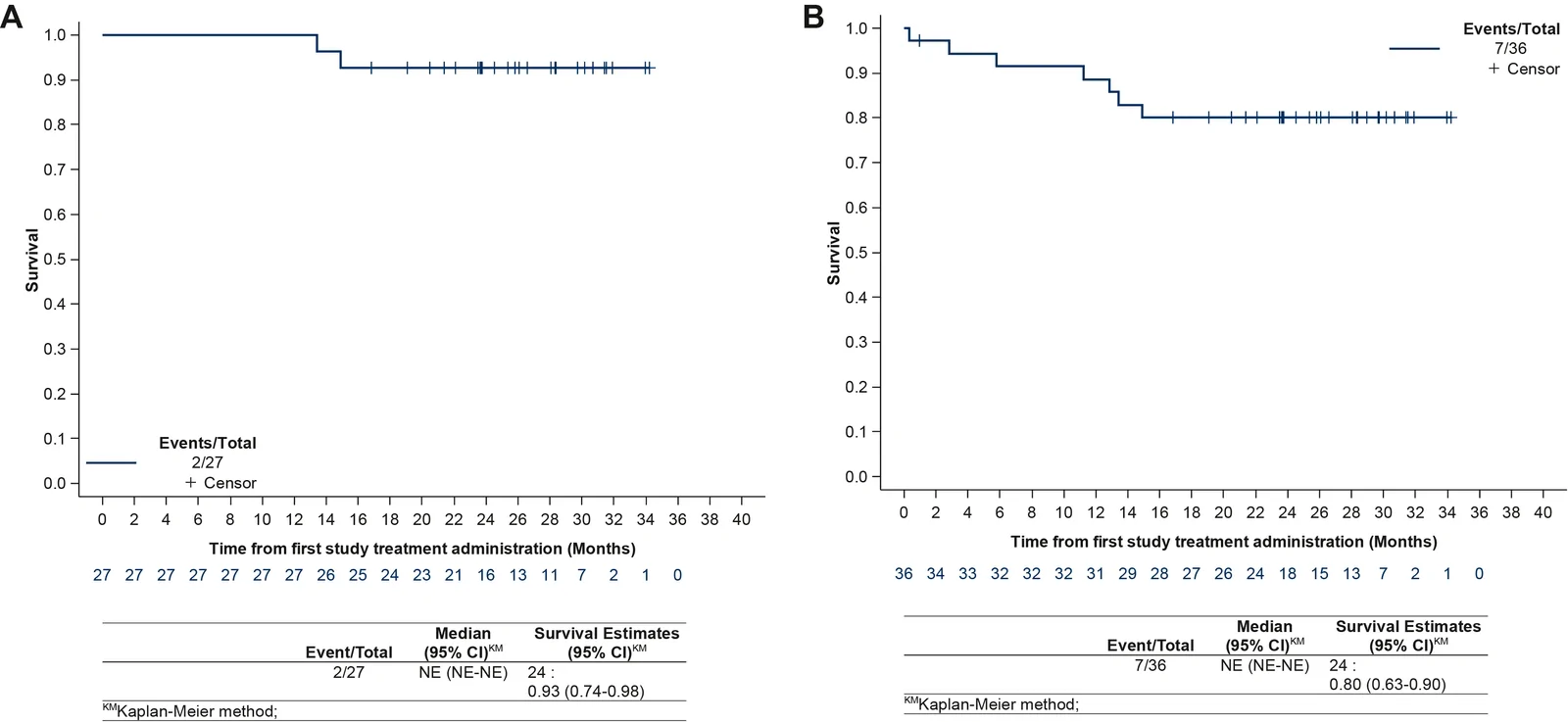
**Event-free survival (EFS).** (A) EFS in the subgroup of patients with surgery after one or two cycles of pembrolizumab (*N* = 27); (B) in all the patients after one or two cycles of pembrolizumab, operated and nonoperated (*N* = 36).

With a median follow-up of 25.8 (95% CI 23.6-28.3) months, 25 of the 27 operated patients had neither recurrence nor progression or death. In this subgroup of operated patients, the 24-month EFS rate was 93% (95% CI 74% to 98%; Figure 2). Events occurred in only two patients (non–cancer-related death, ypT2N0, *N* = 1; recurrence, ypT3N2, *N* = 1). Among the nine patients who were not operated, five patients experienced disease progression (time to progression 0.3, 2.8, 5.8, 11.3, and 12.8 months, respectively); three patients with early response finally achieved complete tumor regression after 8, 9, and 9 pembrolizumab infusions, respectively; and one had stable disease after one pembrolizumab infusion that was subsequently stopped for toxicity (Tr-pneumopathy). The median overall survival was not reached (two patients with progressive disease died).

In the safety population (*N* = 41), 26 patients experienced TrAEs. A total of 13 grade 3 TrAEs occurred in nine patients (diarrhea; hepatitis; polymyalgia rheumatica; fatigue and general health deterioration; vomiting; weight decreased; metabolism disorders/cell death; ulcerative keratitis; Tr-nephritis; and Tr-pneumonitis). No pembrolizumab-related grade 5 AEs were observed.

## Discussion

This prospective study confirms the feasibility of perioperative pembrolizumab in patients with localized dMMR/MSI EGA. The treatment was well tolerated, with no unexpected safety signals or perioperative complications, despite the high median age (74 years) in our cohort, consistent with the known epidemiology of dMMR/MSI EGA. The pCR rate of 14.8% reported after one or two cycles of pembrolizumab (400 mg flat dose every 6 weeks) was lower than anticipated, likely due to short treatment duration. Indeed, accumulating evidence suggests that 6-month duration of PD-1/programmed death-ligand 1 (PD-L1) monotherapy may be required, whereas ∼3-month exposure of combination therapy of PD-1/PD-L1 with CTLA-4 inhibitors may be sufficient to achieve complete response in selected patients with EGA.^8^^,^^14^, ^15^, ^16^ Although the pCR rate was modest, disease control outcomes were encouraging, and even a short course of anti-PD-1 monotherapy may confer clinical benefit in a subset of patients with dMMR/MSI EGA. The 24-month EFS rate of 93% in patients who underwent surgery is encouraging, although it should be interpreted with caution due to the small sample size. In the overall population of 36 patients (irrespective of surgical status), the 24-month EFS rate was 80% (95% CI 63% to 90%), suggesting sustained outcomes in the full cohort. The discrepancy between the relatively low pCR rate (14.8%) and the encouraging 24-month EFS of 93% in operated patients raises important questions regarding interpretation. Although neoadjuvant immune checkpoint blockade has yielded high pCR rates in dMMR CRCs—67% in dMMR tumors in the NICHE-2 trial^7^ and 60% in the colorectal cohort of the IMHOTEP trial^17^—the lower pCR rate observed in the esophagogastric cohort may reflect tumor site-specific biology and/or insufficient exposure to neoadjuvant treatment rather than questioning the relevance of pCR as a surrogate endpoint in this setting. Lower clinical response rates in esogastric compared with colorectal adenocarcinomas were also reported with dostarlimab.^8^ The specific contribution of post-operative pembrolizumab to sustain disease control, in addition to neoadjuvant pembrolizumab in 17 of the 27 operated patients in the IMHOTEP study, remains an open question.

In this context, the phase III trial ATOMIC demonstrated a significant disease-free survival benefit with adjuvant atezolizumab in resected stage III dMMR colon cancer.^18^ Although conducted in a different disease setting, these findings support the hypothesis that post-operative immune checkpoint blockade may provide clinically meaningful benefit independently of pCR achievement. Whether the favorable outcomes observed in IMHOTEP are related to neoadjuvant priming, adjuvant pembrolizumab exposure, or both, warrants further investigation.

Over the last 5 to 7 years, the therapeutic landscape of EGA has undergone profound changes, both in early-stage and advanced disease. Among the most significant advances, the introduction of ICIs and the identification of new targets such as Claudin 18.2 enabled the development of targeted therapies. Understanding of the distinct molecular behavior of dMMR/MSI tumors also represented a major milestone in the clinical management of EGA. In 2017, the *post hoc* analysis of the MAGIC trial demonstrated that perioperative epirubicin–cisplatin–5-fluorouracil chemotherapy did not benefit patients with dMMR/MSI tumors compared with surgery alone, leading to a median overall survival of 9.6 compared with 22.5 months (hazard ratio 2.22, *P* = 0.04).^2^

The individual patient data meta-analysis of four randomized clinical trials (MAGIC, CLASSIC, ARTIST, and ITACA-S), totaling 121 patients with dMMR/MSI EGA, confirmed the absence of benefit of perioperative or adjuvant chemotherapy in this molecularly defined subgroup.^3^ The two nonrandomized phase II studies—NEONIPIGA and INFINITY—investigating dual immune checkpoint blockade in patients with dMMR/MSI EGA^8^^,^^9^ reported promising pCR rates of 59% in 29 operated patients and 60% in 15 operated patients, respectively. In addition, *post hoc* analyses from two recent phase III trials assessing perioperative chemo-immunotherapy in resectable EGA showed pCR rates of 12.9% (KEYNOTE-585, NCT03221426) and 28% (MATTERHORN, NCT04592913) in MSI subgroups after 2 months of neoadjuvant chemotherapy and PD-1/PD-L1 inhibitor combination, questioning a potential deleterious effect of chemotherapy on ICI efficacy in localized dMMR/MSI EGAs.^19^, ^20^, ^21^ Exploratory data from the phase II part of the DANTE trial reported higher pCR rates with atezolizumab added to perioperative FLOT than with FLOT alone, the magnitude being greater in the 23 patients with MSI tumors (atezolizumab–FLOT: 63%; FLOT alone: 27%). Whether outcomes could be improved with combination strategies (either with another ICI or chemotherapy) or via extended treatment duration remains an open question.

Some limitations need to be underlined in our study, notably the occurrence of some post-enrollment exclusions, the choice leading investigators to administrate one or two neoadjuvant pembrolizumab infusions, the rate of patients treated and finally not operated—such situations reflect current realities in routine clinical practice commonly reported in single-arm trials.

The results of the IMHOTEP study contribute to the evolving therapeutic landscape by introducing a novel approach focused on minimized neoadjuvant treatment exposure. Notably, among neoadjuvant studies conducted to date, IMHOTEP is the first trial evaluating anti-PD-1 monotherapy administered over a 6- to 12-week window before surgery. This strategy aims to be both realistic and scalable, aligning with current surgical workflows and timelines. This approach may be particularly relevant considering that patients with MSI EGA are generally older than those with MSS disease and may offer a potential alternative to current standard-of-care perioperative strategies such as durvalumab–FLOT. Finally, whether a nonoperative management strategy in dMMR/MSI EGA can be proposed is open to debate. First results issued from the INFINITY study (cohort 2) showed that a nonoperative management strategy may be successful in 70% of patients, with cautious interpretation considering the limited sample size (*N* = 18) and short median follow-up (8 months). Clinical trials are still ongoing (NCT06059495, NCT06250036).

### Conclusion

Perioperative pembrolizumab monotherapy showed lower pCR rates than expected but encouraging 2-year EFS results. Perioperative pembrolizumab can be considered as a safe and feasible treatment for localized dMMR/MSI esophagogastric cancers. Several questions remain unanswered in this setting, questioning the need for combination and the optimal neoadjuvant treatment duration.
