# Supplementary material for: Is pembrolizumab as safe and efficient as neoadjuvant treatment in localized dMMR/MSI esophagogastric cancers? Results from the IMHOTEP phase II study
Source: ESMO Open. 2026 Sep 9;11(9):108343. doi: 10.1016/j.esmoop.2026.108343 (PMC13582005; doi:10.1016/j.esmoop.2026.108343)
Supplement: Supplementary file 1 [file mmc1.docx]

## **Supplementary methods S1**

## **Inclusion criteria**

I1. Age ≥18 years on the day of signing informed consent.
I2. Histologically proven locally advanced non-metastatic tumor included in one of the following cohorts:
    - Colon or rectal cancer (cT3/T4 N0 M0 ou cT N+ M0) OR
    - **Oesogastric (gastric, gastro-oesophageal or oesophageal) cancer (cT2 to cT4 N M0)** OR
    - Endometrial carcinoma (stage III) OR
    - Other tumor types (cT2 to cT4 N M0): small bowel adenocarcinoma (duodenum, jejunum, ileum).
I3. MSI/dMMR status established by immunohistochemistry (IHC) for MMR protein expression **and** polymerase chain reaction (PCR) [*both techniques are required]* and validated by coordinator’s team.

*MMR and/or MSI tumors will be assessed using IHC with four antibodies (anti-MLH1, anti-MSH2, anti-MSH6, and anti-PMS2) and PCR (pentaplex panel is recommended: BAT-25, BAT-26, NR-21, NR-24, and NR-27) prior to screening. Loss of MLH1 and PMS2 /or MSH2 and MSH6 / or MSH6 alone / or PMS2 alone protein staining by IHC indicates dMMR, and tumor with ≥ 2 unstable markers analyzed on PCR proves MSI/dMMR.*

**OR EBV-positive gastric cancers.** EBV positivity will be assessed by EBER (EBV-encoded small RNAs) in situ hybridization (ISH) (EBER-PNA EnVision flex probe, Dako)). The intensity of staining (weak, moderate or intense) and the percentage of positive cells will be recorded. Cases showing nuclear staining in at least 5% of tumour cells will be considered positive for EBV infection.

I4. Eastern Cooperative Oncology Group Performance status (ECOG-PS) 0 to 1 within 7 days prior to the inclusion.
I5. Adequate bone-marrow, hepatic, and renal functions, within 10 days prior to the start of study treatment, with:
    - Hemoglobin ≥9g/dl or ≥ 5.6mmol/l, Neutrophils ≥1.0 x 10^9^/l, Platelets ≥100x10^9^/l,
    - Creatinine ≤1.5 x upperl limit of normal (ULN) OR Calculated creatinine clearance ≥50 ml/min/1.73m² using either the MDRD or CKD-EPI formula,
    - Aspartate aminotransferase (AST) and Alanine aminotransferase (ALT) ≤3xULN, total bilirubin ≤1.5xULN (OR direct bilirubin ≤ULN for patients with total bilirubin levels >1.5×ULN).
    - International normalized ratio (INR) OR prothrombin time (PT) ≤1.5×ULN unless participant is receiving anticoagulant therapy as long as PT or activated partial thromboplastin time (aPTT) is within therapeutic range of intended use of anticoagulants.
I6. Covered by a medical/health insurance.
I7. Willingness and ability to comply with scheduled visits, treatment plans, laboratory tests, and other study procedures.
I8. Patients of childbearing potential willing to use effective contraceptive measures or abstain from heterosexual activity for the course of the study through 4 months after the last dose of pembrolizumab MK-3475 adjuvant treatment or 6 months after adjuvant chemotherapy or being surgically sterile.
I9. Signed and dated IRB/IE approved informed consent form.

## **Non-inclusion criteria**

E1. MSS/pMMR tumors.
E2. Metastatic disease (stage IV).
E3. HIV positive with CD4 count under 400 cells/mm^3^.
E4. Active Hepatitis B virus (HBV), defined by a positive hepatitis B surface antigen [HBsAg] test prior to inclusion and HBV DNA >2000 IU/ml or hepatitis C virus (HCV) infection (HCV RNA >10^3^copy/ml and positive anti-HCV antibodies).
E5. Active systemic autoimmune disease that has required systemic treatment in the past 2 years (i.e. with use of disease modifying agents, corticosteroids or immunosuppressive drugs). Replacement therapy (e.g., thyroxine, insulin) is not considered a form of systemic treatment and is allowed.
E6. Interstitial lung disease.
E7. Prior (non-infectious) pneumonitis requiring systemic corticosteroid therapy or current pneumonitis.
E8. History of severe hypersensitivity to another monoclonal antibody.
E9. Receiving immunosuppressive therapy or having received corticosteroids (in dosing exceeding 10 mg daily of prednisone equivalent) within the last 2 months before inclusion.
E10. Active infections.
E11. Live vaccine within 30 days prior to the first dose of study drug. Examples of live vaccines include, but are not limited to, the following: measles, mumps, rubella, varicella/zoster (chicken pox), yellow fever, rabies, Bacillus Calmette–Guérin (BCG), and typhoid vaccine. Seasonal influenza vaccines for injection are generally killed virus vaccines and are allowed; however, intranasal influenza vaccines (eg, FluMist®) are live attenuated vaccines and are not allowed.
E12. Known history of active TB (Bacillus Tuberculosis)
E13. Known psychiatric or substance abuse disorders that would interfere with cooperation with the requirements of the study.
E14. Pregnant or breastfeeding woman or patient expecting to conceive or father children within the projected duration of the study, starting with the screening visit through 4 months after the last dose of study treatment.
E15. Patient requiring tutorship or curatorship.
E16. Ongoing anti-cancer treatment for another cancer (to be discussed with the coordinator in case of hormone therapy).

**Supplementary figure S2.** **Schematic representation of the patients treated with one or two cycles of pembrolizumab, operated and non operated (N=36)**
